# Culture-Independent Study of the Late-Stage of a Bloom of the Toxic Dinoflagellate *Ostreopsis* cf. *ovata*: Preliminary Findings Suggest Genetic Differences at the Sub-Species Level and Allow ITS2 Structure Characterization

**DOI:** 10.3390/toxins7072514

**Published:** 2015-06-30

**Authors:** Vitor Ramos, Daniele Salvi, João Paulo Machado, Micaela Vale, Joana Azevedo, Vitor Vasconcelos

**Affiliations:** 1 CIIMAR/CIMAR—Interdisciplinary Centre of Marine and Environmental Research, University of Porto, Rua dos Bragas 289, 4050-123 Porto, Portugal; E-Mails: vtr.rms@gmail.com (V.R.); jprmachado@gmail.com (J.P.M.); mica.vale@gmail.com (M.V.); joana_passo@hotmail.com (J.A.); 2 CIBIO, Centro de Investigação em Biodiversidade e Recursos Genéticos, InBIO, Universidade do Porto, Campus Agrário de Vairão, 4485-661 Vairão, Portugal; E-Mail: danielesalvi.bio@gmail.com; 3 Departamento de Biologia, Faculdade de Ciências, Universidade do Porto, Rua do Campo Alegre, 4169-007 Porto, Portugal

**Keywords:** *Ostreopsis*, HAB, culture-independent approach, phylogeny, ITS2 secondary structure, NE Atlantic, Portugal, environmental samples

## Abstract

Available genomic data for the toxic, bloom-forming, benthic *Ostreopsis* spp. are traditionally obtained from isolates rather than from individuals originally present in environmental samples. Samples from the final phase of the first reported *Ostreopsis* bloom in European North Atlantic waters (Algarve, south coast of Portugal) were studied and characterized, using a culture-independent approach. In the first instance, a microscopy-based analysis revealed the intricate complexity of the samples. Then, we evaluated the adequacy of commonly used molecular tools (*i.e.*, primers and nuclear ribosomal markers) for the study of *Ostreopsis* diversity in natural samples. A PCR-based methodology previously developed to identify/detect common *Ostreopsis* species was tested, including one new combination of existing PCR primers. Two sets of environmental rRNA sequences were obtained, one of them (1052 bp) with the newly tested primer set. These latter sequences encompass both the ITS1-5.8S-ITS2 region and the D1/D2 domain of the LSU rRNA gene, leading us to an accurate identification of ITS2. In turn, this allowed us to predict and show for the first time the ITS2 secondary structure of *Ostreopsis*. With 92 bp in length and a two-helix structure, the ITS2 of this genus revealed to be unique among the dinoflagellates. Both the PCR approach as the phylogenetic analyses allowed to place the *Ostreopsis* cells observed in the samples within the *O.* cf. *ovata* phylospecies’ complex, discarding the presence of *O.* cf. *siamensis*. The (phylo)genetic results point out a certain level of nucleotide sequence divergence, but were inconclusive in relation to a possible geographic origin of the *O.* cf. *ovata* population from the Algarve’s bloom.

## 1. Introduction

The harmful algal bloom (HAB)-forming *Ostreopsis ovata* Fukuyo and *Ostreopsis siamensis* Schmidt are toxic, benthic (including epibenthic, epiphytic, or tychoplanktonic) dinoflagellates species now recognized as being distributed from tropical to temperate coastal waters [1], with increasingly frequent appearances in different regions of the Mediterranean basin [2]. These two species are known to co-occur [3,4] although their morphological differentiation is quite difficult. They are seen as cryptic species [5] and their morphological identification requires scanning electron microscopy (SEM) analysis of their thecal plate arrangement and morphology and location of sulcal plates [6,7,8]. They often form blooms under favorable environmental conditions [9], which can either be easily visible or remain undetected underwater [10]. The recently increasing frequency of this ecological phenomenon in the Mediterranean Basin [1,2], along with the toxic compounds produced by these dinoflagellates [11], makes them likely to pose direct or indirect health risks to bathers [12], seafood consumers [13] and may also endanger important marine biocenoses [11]. So far, the large majority of bloom events in Europe have occurred along the Mediterranean coastline and were largely dominated by *O. ovata* [1]. However, low densities of co-occurring *O. ovata* and *O. siamensis* have also been recorded in the Atlantic waters of the Iberian Peninsula [14,15,16,17]. In the north-eastern Atlantic Ocean region, the Bay of Biscay (Cantabrian Sea, Spain/France) is the northernmost location where *Ostreopsis* was already detected [15,17]. The occurrence of *O.* cf. *siamensis* and/or *O.* cf. *ovata* in Portugal (including Azores and Madeira Islands) is known from monitoring studies and anecdotal reports [14,16,17,18] or from the existence of isolates collected in Portuguese waters [19,20].

The Iberian Atlantic coast adjacent to the Mediterranean Sea is a warm-temperate region with a marked seasonality, where upwelling events are frequent. Within this region, the western coast of Algarve—the most important sun and beach destination of Portugal—has topographic and geomorphologic features and hydrodynamic and environmental conditions (e.g., wave action, temperature) suitable for the emergence and development of *Ostreopsis* spp. blooms [9,21,22]. Actually, the first (and so far the only) known bloom of an *Ostreopsis* species in the Atlantic coast of continental Europe was recorded in this region, in September 2011 [23].

The microscopy-based identification and discrimination of *Ostreopsis* species present in the field samples is difficult given the morphological resemblance between species, their morphological plasticity and consequent high cell-size variability [4,24,25,26], and the possible co-occurrence of *Ostreopsis* spp. in the natural environment [3,24]. Since thorough morphological identifications at the species level require SEM analyses [6,7], rapid and reliable molecular-based methodologies were developed to distinguish *Ostreopsis* species [5,27]. The most widely and successfully used molecular markers to identify dinoflagellates belong to the nuclear ribosomal RNA gene cluster [27,28,29,30]. The majority of the *Ostreopsis* spp. nucleotide sequences available in public databases include either the ITS1-5.8S-ITS2 rRNA region [19,24,31,32] or the variable domains of the 28S rRNA gene [19,32], and were mainly obtained from cultured isolates.

After being alerted by the news [33] that the presence of *Ostreopsis* led to a prohibition of bathing in Algarve (south of Portugal), we decided to adopt a culture-independent approach to study and characterize floating clumps derived from this 2011 bloom event (see also [23]). Samples were collected in three affected beaches, coinciding with the last two days of bathing prohibition. The algal composition of the samples proved to be more diverse and intricate than initially appreciated. Available molecular tools for the study of *Ostreopsis* in field samples were tested, and their feasibility evaluated. Albeit the complex composition of the samples, we were able to obtain two sets of rRNA sequences from naturally occurring *Ostreopsis* cf. *ovata*. One of these sets was achieved through a new combination of existing PCR primers, which has the advantage of covering four different rRNA loci in a single PCR amplification. Additionally, we were able to define the boundaries of ITS2 of *Ostreopsis*, allowing the prediction of its secondary structure.

## 2. Results

### 2.1. Morphometric Analysis and Microalgal Community

**Figure 1:**
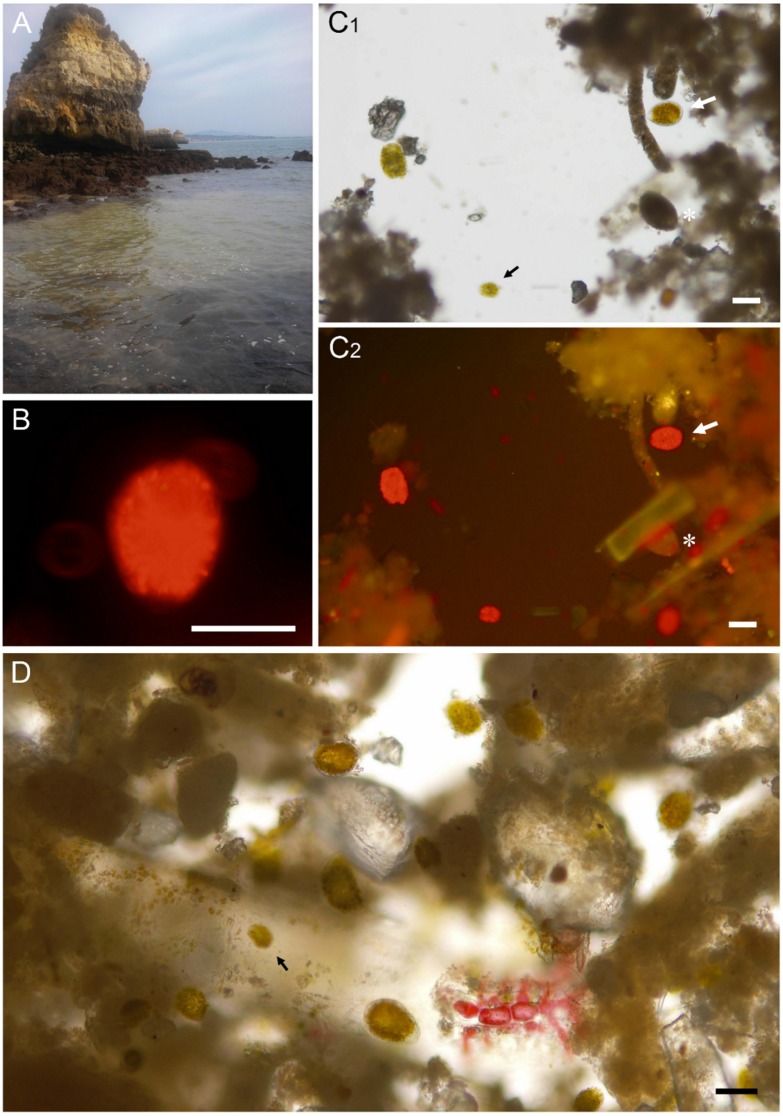
Photograph (**A**) and micrographs (**B**–**D**) of the bloom. (**A**) Greenish-brown water discoloration at Camilo; (**B**) Red autofluorescence of chloroplasts radially-disposed in an *Ostreopsis* cf. *ovata* cell by epifluorescence microscopy; (**C**) The tear-shaped *Ostreopsis* cf. *ovata* cells were evident under light microscopy (e.g., **C_1_**, white arrow), however, epifluorescence microscopy (**C_2_**) allowed to detect other chlorophyll-containing organisms (pseudo-colored in red), including unidentified cysts (**C_1_** and **C_2_**, asterisk), which otherwise could go unnoticed; (**D**) This later-staged, detached bloom was found to be a very complex sample with sediment, detritus, and different plankton and benthic (both phyto- and zoo-) organisms embedded in a mucilaginous network. The “small cell” morphotype [4,25,26] of *Ostreopsis* was also observed (**C_1_** and **D**, black arrow). Scale bars: 50 μm.

Cells assigned to *Ostreopsis* sp. were ovoid to teardrop-shaped in dorsoventral (DV) view, with many golden (brownish) chloroplasts radially disposed (Figure 1B,C). They were variable in size (Table 1 and Figure 1D) and presented an average DV diameter of 73 μm and 51 μm in width (W), ranging between 42–105 μm and 32–75 μm, respectively (*n* = 45). The DV/W ratio for average values was 1.43. Morphometric values presented by the Algarve’s *Ostreopsis* cells were comparable with those from environmental or cultured *Ostreopsis* cf. *ovata* and *Ostreopsis* cf. *siamensis* cells obtained from other studies (Table 1).

Small cells [4,25,26] and Type A thin-walled cysts [25] morphotypes were identified (Figure 1C,D and Figure S1, respectively) and assigned to *Ostreopsis*. However, other possible *Ostreopsis* cysts (e.g., Figure 1C) morphologically difficult to distinguish [4,25,26] were accounted as unidentified (Table 2). The same was applied to morphotypes [25] that couldn’t be undoubtedly assigned to *Ostreopsis* (see also notes in Table 1).

**Table 1:** Morphometric features of *Ostreopsis* cells from the Algarve’s bloom and comparison with *O. ovata* and *O*. cf. *siamensis* measurements from other studies. The values are range measurements (μm) of dorsoventral (DV) and anterioposterior (AP) diameters, and width (W).

Taxa (type of sample)	DV	W	AP	DV/W ratio	DV/AP ratio	Reference
*Ostreopsis*	42–105	32–75	-	1.4	-	This study
*O*. cf. *ovata* (environmental)	26–62	13–48	14–36	1.8	~2	[3]
*O*. cf. *ovata* (environmental)	19–75	13–60	10–31	-	2.3	[4]
*O*. cf. *ovata* (culture) ^a^	55–84	30–62	-	1.2–2	-	[17]
*O*. cf. *ovata* (culture) ^b^	40–60	31–49	-	1.3	-	[25]
*O*. cf. *ovata* (culture)	27–65	19–57	-	-	<2	[24]
*O*. cf. *ovata* (culture)	27–85	17–65	-	-	-	[26]
*O*. cf. *siamensis* (environmental)	36–66	24–50	14–26	~1.4	~3	[3]
*O*. cf. *siamensis* (environmental)	50–75	38–62	-	~1.3	>4	[24]
*O*. cf. *siamensis* (culture) ^a^	52–75	27–57	-	1.1–2.1	-	[17]

^a^ Specimens collected from Atlantic Iberian Peninsula waters; ^b^ Values are for the entire culture population; in addition to vegetative cells, Accoroni *et al.* [25] distinguished seven other categories (*i.e.*, morphotypes) of *O*. cf. *ovata* cells within the population, including three different types of cysts.

**Table 2:** Microalgal abundance in the water column samples, at the three studied sites.

Taxa	Number of taxa (cells∙L^−1^) per sampling site ^a^		
	Burgau	Luz	Camilo
*Ostreopsis* sp.	2520 (13.1%)	5480 (19.8%)	13,040 (18.3%)
Other dinoflagellates ^b^	5880 (30.6%)	200 (0.7%)	1520 (2.1%)
Diatoms	5520 (28.7%)	5840 (21.1%)	53,040 (74.4%)
Cyanobacteria	2600 (13.5%)	10,880 (39.3%)	-
Other microalgae	2720 (14.1%)	5280 (19.1%)	3680 (5.2%)
Total	19,240	27,680	71,280

^a^ numbers within brackets represents relative frequencies × 100; ^b^ includes unidentified dinoflagellates/cysts.

Bloom was visually evident only in one of the three sampling locations (Figure 1A), at the time of water collection. In spite of this, *Ostreopsis* cells represented a major portion of the microalgal community of all three seawater samples (Table 2). In Burgau, the local where *Ostreopsis* was shown to be less abundant (2520 cells·L^−1^) in the water column, they corresponded to a third of the dinoflagellates present in the sample, representing 13.1% of the total microalgal community. The other dinoflagellates species observed belonged to several genera such as *Alexandrium*, *Ceratium*, *Dinophysis*, *Prorocentrum*, *Protoperidinium*, without dominance of any of them. In Luz and Camilo (with 5480 and 13,040 cells·L^−1^, respectively) *Ostreopsis* accounted to the large majority (>89% and >96%, respectively) of the dinoflagellates. However, its relative abundance within the overall microalgae community was 19.8% in Luz and 18.3% in Camilo.

### 2.2. Testing of PCR Primer Sets

**Figure 2:**
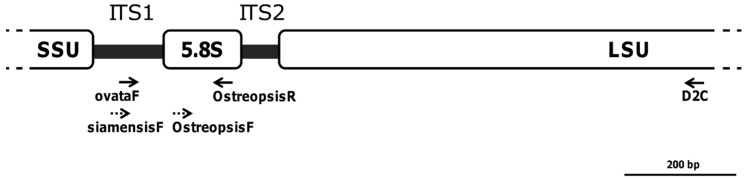
Schematic diagram of the rRNA gene cluster showing the binding sites of the PCR primers used in the study (roughly drawn to scale). Dashed arrows represent primers for which an amplification product was not obtained or the sequencing results of the amplicons were not considered afterward (see text for detail).

The only primer pair tested that gave no PCR amplicons was the species-specific set siamensisF/OstreopsisR (see Figure 2 and Table S1). Due to its shortness (<50 bp after removal of primer sequences) and redundant rRNA coverage (Figure 2), amplicons amplified by the genus-specific primer pair (OstreopsisF/OstreopsisR) were discarded from further analyses. However, the specificity of the PCR amplification was examined and confirmed by sequencing (data not shown). All remaining sequences retrieved from the clone libraries were amplified with the primer sets ovataF/OstreopsisR or ovataF/D2C. In accordance with the PCR results, BLASTn searches performed in GenBank showed that all the clone sequences belonged to the phylotype *Ostreopsis* cf. *ovata* (similarity ≥ 99%).

### 2.3. Phylogenetic and Genetic Analyses

**Figure 3:**
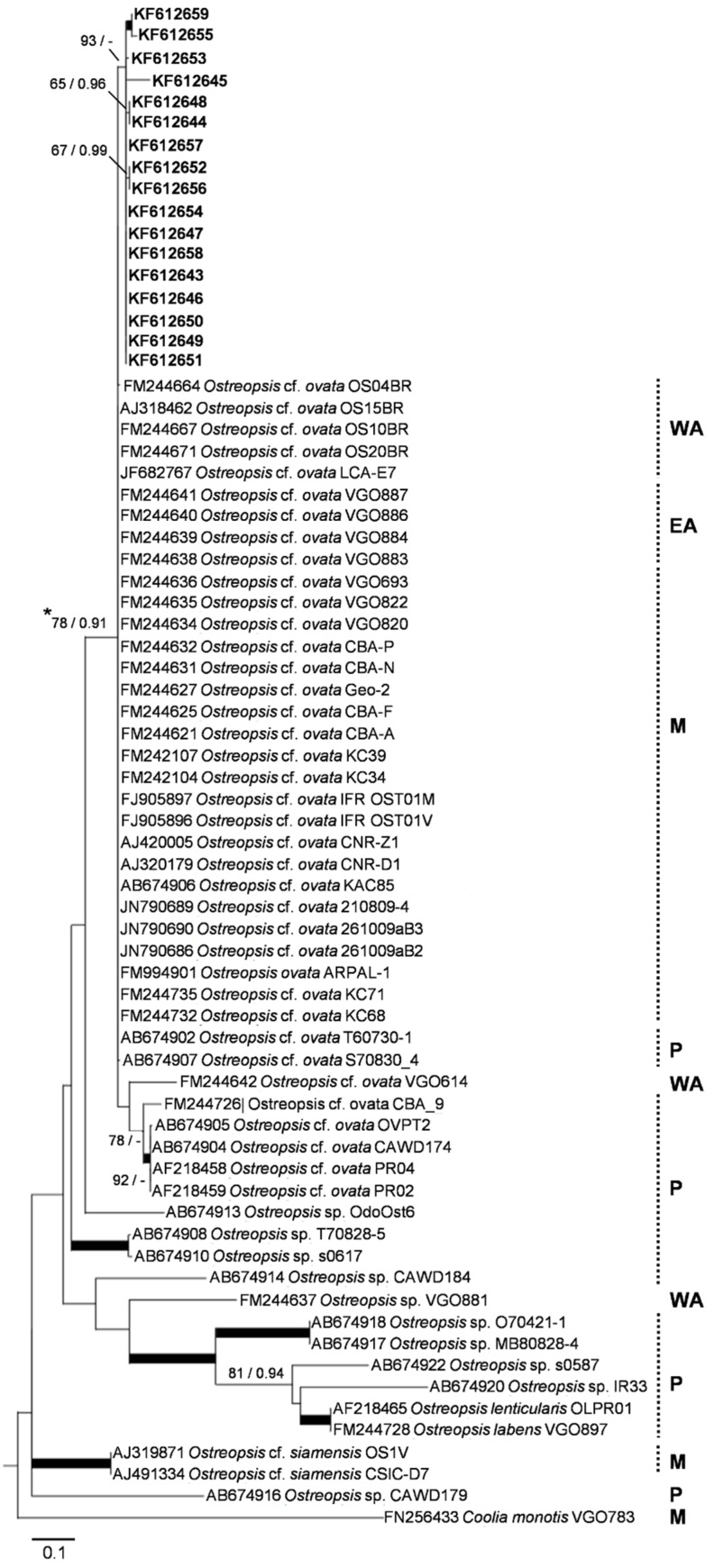
Maximum likelihood tree (−lnL = 2786.27) of partial ITS1-5.8S-ITS2 rRNA region sequences from *Ostreopsis* spp. isolates and from environmental clones obtained in this study (in bold). The dataset comprised 70 sequences, with 219 unambiguously-aligned nucleotide sites. The asterisk indicates the node of the *Ostreopsis* cf. *ovata* clade/species complex *sensu* Sato *et al.* [32] (after [19]). Nodal support values indicated near internal branches were determined by ML and BI methods, respectively; bootstrap values (for ML) below 65% and posterior probability values (for BI) below 0.80 were omitted. Thick lines indicate simultaneous ≥ 85% bootstrap values and ≥ 0.95 posterior probability support for tree branches. Scale bar represents 0.1 nucleotide substitutions per site. Legend: M—isolates collected in the Mediterranean Sea region, EA—East Atlantic, WA—West Atlantic, P—Pacific.

All the Algarve’s sequences were placed in a clade with sequences of *Ostreopsis* cf. *ovata* isolates collected from different regions of the world (Figure 3 and Figure S2). In the phylogenetic trees derived from ITS1-5.8S rRNA region (Figure 3) the clone sequences grouped in a single cluster with a good ML bootstrap value (93%) but lacking BI posterior probability support. Despite more than half of the sequences of this cluster revealing high genetic similarity (>99%), some of the clone sequences were phylogenetically slightly different from the others. In fact, three groups of them clustered together as tight sub-clusters (Figure 3). This contrasts with the large amount of isolates that belong to the same *Ostreopsis* cf. *ovata* clade, which have identical sequences (representing a ribotype, *sensu* Sato *et al.* [32]). A similar phylogenetic pattern was obtained for the dataset with the larger sequences (ITS1-5.8S-ITS2-LSU region) (Figure S2). In this case, the environmental clone sequences did not group in a single distinct clade, but, as seen in Figure 3, several sequences were grouped into sub-clusters with good bootstrap support, even if phylogenetically extremely close. With respect to this last dataset, the analysis of sequence divergence performed (Table S2) shows that Algarve’s sequences present slightly higher genetic variability values than those from other groups of phylogenetically close sequences. When compared to the other geographically established groups, the sequences from the Algarve’s bloom revealed the highest intra-group divergence (Table S2, in bold), in all the three rRNA regions analyzed (0.003 base substitutions per site for the ITS1-5.8S rRNA gene fragment, 0.009 for ITS2, and 0.009 for LSU). Comparisons across columns, in Table S2, show that these values are even higher than any inter-group divergence (plain characters), for each locus. Similar findings were obtained for base differences per sequence (values within brackets, in Table S2).

### 2.4. ITS2 Secondary-Structure Model of Ostreopsis cf. ovata

**Figure 4:**
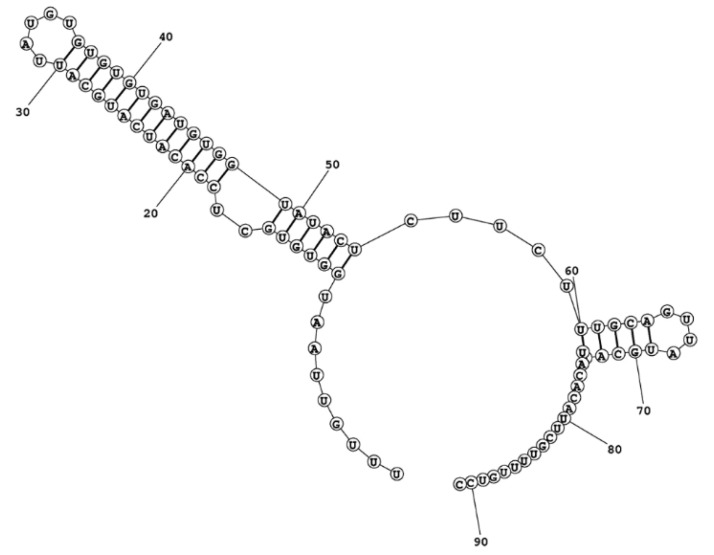
Predicted ITS2 rRNA secondary structure model of *Ostreopsis* cf. *ovata* derived from the free energy minimization and the maximum expected accuracy approaches.

The sequence length of ITS2 in *Ostreopsis* cf. *ovata* was 92 bp. The ITS2 secondary structure model of *O.* cf. *ovata* derived from free energy minimization algorithm was identical to that obtained with the maximum expected accuracy approach. This model shows a two-helix structure (Figure 4), with the first helix, including 19 base pairs, separated by five single-stranded nucleotides from a short second helix of five base pairs.

The highly divergent ITS2 sequence of *Ostreopsis* compared to *Alexandrium* and *Coolia* made the homology assessment among these taxa unfeasible, preventing both a reliable alignment (see also Sato *et al.* [32]) and the prediction of *Ostreopsis* ITS2 structure based on homology modelling (results not shown).

## 3. Discussion

*Ostreopsis* blooms are characterized by a rapid covering of the benthos, producing more or less extensive mats in shallow and sheltered coastal waters [11,34]. In late bloom phases, the mucilaginous network can either become detached [13,34] and float as clumps on the water column or remain undetectable underwater [10]. The Algarve’s bloom probably had its origin away from the shoreline, in the submerged flat bedrock bottom typical of this region. Afterwards, large portions of the mat may have loosened and unfastened from the substrate and reached several beaches. The bloom has determined a temporary bathing prohibition imposed by the competent authorities [33], which in turn caused negative impacts on the local economy of the affected area. After being made aware of the news about this *Ostreopsis* bloom event [33], we proceed with the sampling campaign. After just a day, the authorities considered that this HAB no longer posed a public health risk, and the prohibition was lifted.

### 3.1. Characterization of the Bloom and Microalgae Composition

Bloom samples (*i.e.*, floating mucilaginous clumps) were surprisingly diverse and intricate in their composition (see Figure 1C,D as an example). The initial presumption was that they would be clearly dominated by *Ostreopsis* cells. However, the light microscopic examination of the samples revealed a complex tycoplanktonic community (Table 2). At most, *Ostreopsis* cells accounted for up to 20% of the total number of microalgae cells observed in one sample. However, the number of *Ostreopsis* cells recognized in each sample may be underestimated due to the presence of several cysts that could not be assigned to any dinoflagellate. Cysts are even more difficult to identify than vegetative cells, particularly in field samples [3,25,26]. For this reason, they were counted as unidentified dinoflagellates (Table 2).

The mucilage typically exuded in large amounts by *Ostreopsis* cf. *ovata* [34] have trapped or provided harbouring for other benthic taxa and/or phytoplankton, such as quick-growing, opportunistic species like diatoms and cyanobacteria (Table 2). These taxa were visibly embedded in the mucus, mixed along with *Ostreopsis* cells. Clumps also harboured detritus and small- to medium-sized zooplankton and macroalgae in earlier stages (as an illustration, see Figure 1D). This broad assortment of organisms and the amount of different cell morphotypes observed (including cysts) [26] are very likely linked to the phase of the bloom. Accoroni *et al.* [4,25] observed that the percentage of some *Ostreopsis* cf. *ovata* morphotypes other than vegetative cells increased at the final phase of blooms. These include, for instance, Type A thin-walled cysts (Figure S1) and dark cells. The same authors also found that the cell size (e.g., DV length) is higher in final/decline phases of blooms. Moreover, while studying Mediterranean’s blooms, they have observed that *Ostreopsis* cf. *ovata* begin to decline by the mid or end of September [4].

### 3.2. Morphological Characterization, Molecular Identification, and (Phylo)Genetic Analyses

The morphological analysis performed allowed us to assume that the *Ostreopsis* cells present in the field samples belonged to a single morphospecies. The characteristics observed are in agreement with the description of *O. ovata*, which is much smaller than other *Ostreopsis* species [6,7,34]. The range of morphometric values observed is also in concordance with those found in naturally occurring cells of this species (Table 1) [4,24,26]. Nevertheless, its size range is known to overlap with that of the morphologically similar cells of *O. siamensis* (see also Table 1). Moreover, this species is sometimes seen to co-occur with *O. ovata* in Mediterranean waters [3,24] and its occurrence was already described for mainland Portugal [14]. Likewise, the assumption of the occurrence of a single *Ostreopsis* morphospecies lacked firm support. David *et al.* [23], which have studied the same bloom event, had the same difficulties in appraising the presence of one or both species, even after analysing details of the thecal plates. One useful morphometric parameter that seems distinct between the two species is the DV/AP ratio [4] (see Table 1). However, the measurement of the anterioposterior diameter better requires SEM analysis. Therefore, the arrival of rapid and reliable molecular-based approaches to distinguish *Ostreopsis* species in natural samples [5,27] was so appreciated, particularly for monitoring purposes. In fact, the results from the molecular methodology here adopted have supported the presence of only one taxon. The presence of *O. siamensis* could be discarded by PCR analysis, while the molecular identification and the phylogenetic analysis (Figure 3) allowed to assign all the clone sequences to the phylospecies *O.* cf. *ovata* [19].

Several isolates obtained from different world’s marine regions are known to belong to the *O.* cf. *ovata* clade/species complex. Two independent phylogeographic researches [19,32] on the distribution of *Ostreopsis* species have extensively studied those isolates. Our phylogenetic analysis (Figure 3) did not allow us to discern about a possible biogeographic origin for the *O.* cf. *ovata* population from the Algarve’s bloom. The clone sequences obtained were similarly distant (Figure 3) and divergent (Table S2) from isolates collected in either the Western or Eastern Atlantic Ocean, the Mediterranean Sea, or those collected in the Pacific region. These findings are in consonance with the phylogeographic studies from Penna *et al.* [19] and Sato *et al.* [32]. Moreover, we have observed some genetic variations within the *O.* cf. *ovata* environmental sequences from Algarve, at a level unobserved within any of the aforementioned geographic groups of isolates (Table S2). The ITS2 and LSU groups of sequences from the Algarve’s bloom have shown the highest number of nucleotide substitutions per site, both with a value of 0.009 (Table S2). For instance, 0.009 was also the value observed for the overall divergence among all the ITS2 sequences tested, within the sub-cluster. And for the LSU gene sequences, the overall divergence was only 0.004. This is more relevant if we bear in mind that the genetic variability observed, even if minor, is reported in *Ostreopsis* cells belonging to a same bloom event. For instance, the “not so variable” closely related isolates from the Mediterranean group (Table S2) were collected in different populations, in different regions of the Basin. However, these values are well below those observed for the nucleotide divergence within the out-group (sequences from two phylogenetically more distant *O.* cf. *ovata* isolates) or between the out-group and any in-group.

Thus, the amount of variability observed within the Algarve’s sequences population, even if referring to the same phylospecies, is likely to encompass different strain genotypes (*i.e.*, sub-species level). Future studies on this and additional bloom populations sampled through the (phylo)species range may help test this hypothesis.

### 3.3. Evaluation of Oligonucleotides

Our results confirm the suitability of the primers developed by Penna *et al.* [27] for the genus- and species-specific identification (Table S1) of this harmful alga in such complex samples, which harbored numerous other dinoflagellates (Table 2). Among the primer sets tested, the newly combination ovataF/D2C has the advantage of covering four different rRNA loci at one time (Figure 2), making it advisable for phylogenetic studies. The widely used D2C and its original antisense D1R were designed to amplify the 28S rRNA gene of dinoflagellates, in general [35]. Remarkably, even in a sample for which *O.* cf. *ovata* cells were less abundant than cells from all the other dinoflagellates (at Burgau; see Table 2), the new primer pair combination was able to discriminate them. Since the reverse primer D2C did not return any *Ostreopsis* sequence when in combination with D1R, the primer ovataF [27] is likely to be sufficient to ensure the specificity of the PCR reaction.

### 3.4. Considerations on the ITS2 Characterization

The two rRNA internal spacers (ITS1 and ITS2) were proposed as good markers candidates for DNA barcoding of dinoflagellates [30], capable of revealing hidden diversity among cultured isolates [29]. However, *Ostreopsis* was not included in such evaluation [30]. Moreover, available ITS1 and ITS2 sequences are often incomplete and/or incorrectly annotated [36]. For these reasons, we have planned an accurate identification of ITS2, which was fully covered in our ITS1-5.8S-ITS2-LSU sequences. With 92 bp in length, the ITS2 of *Ostreopsis* cf. *ovata* is the shortest among dinoflagellates. This length is in accordance with Pin *et al.* [31], who have estimated an ITS2 size between 87 and 92 bp for *Ostreopsis ovata*. Typically, other dinoflagellates show an ITS2 length of 200–250 bp [29], ranging from 114 bp in *Coolia* [37]—a close genus of *Ostreopsis* [24]—to almost 700 bp in *Amyloodinium* [29]. *Ostreopsis* and *Coolia* belong to Gonyaulacales, an order that shows the fastest evolutionary rate among rRNA genes in dinoflagellates. And, within this order, *Ostreopsis*is the fastest-evolving genus [28]. Moreover, it is well known that the noncoding internal transcribed spacers (*i.e.*, ITS1 and ITS2) have a faster evolutionary rate. Thus, the unusually short ITS2 length of *Ostreopsis* may be a result of such rapid evolution.

The ITS2 secondary structure of *Ostreopsis* does not conform the canonical eukaryote “four domains” architecture [38,39,40], which has been observed in the closely related Gonyaulacales genera *Coolia* and *Alexandrium* [37,41]. ITS2 structure variants deviating from the Eukaryote core structure are known in several organisms including algae (e.g., [42]), plants (e.g., [43]) fungi (e.g., [39]), and animals (e.g., [44]), and are expected about 30,000 variants out of the 50,000 available full ITS2 sequences [40]. This suggests that several organisms, including *Ostreopsis*, have species or organism specific solutions for ITS2 processing [40].

Finally, several properties of the dinoflagellate ITS2 rRNA sequences make this marker a promising molecular tool for species identification but not for phylogeny estimations. Indeed, the extensive length variation shown by dinoflagellate ITS2s, as well as the high sequence divergence observed among genera *Ostreopsis*, *Coolia*, and *Alexandrium* (this study; [29,37]) make the assessment of homology across the Gonyaulacales genera difficult and thus phylogenetic comparison impractical. On the other hand, at a higher level of taxonomic resolution, the relatively low (intra- and inter-) population divergence displayed by dinoflagellate ITS2 sequences (Table S2; [31]) compared to inter-specific differentiation among congeneric species [31], corroborates the utility of the ITS2 as a DNA barcode for the identification/distinguishing of *Ostreopsis* species, as observed in other dinoflagellates [29,30]. In effect, we consider that it can be used as a tool for the (difficult) taxonomy of this genus, which is in need of revision [45]. Future studies will have to explore this appropriateness.

## 4. Experimental Section

### 4.1. Sampling Site Locations, and Samples Collection and Processing

**Figure 5:**
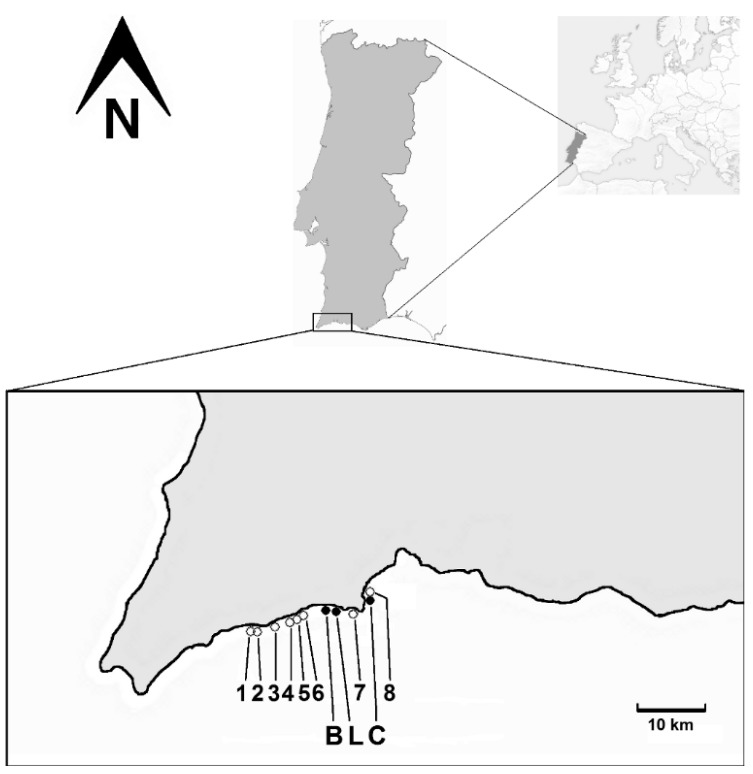
Beaches in the Algarve coast (Portugal’s most touristic destination) affected by the bloom and sampling sites studied (capital letters). Legend: 1, Ingrina; 2, Zavial; 3, Furnas; 4, Salema; 5, Boca do Rio; 6, Cabanas Velhas; 7, Porto de Mós; 8, Dona Ana; B, Burgau (37°04′17.7″ N, 8°46′24.7″ W); L, Luz (37°05′10.1″ N, 8°43′39.3″ W); C, Camilo (37°05′16.7″ N, 8°40′05.4″ W).

One surface seawater sample (depth 0.2–0.3 m below water surface) was collected, using 5 L plastic bottles, in the surf zone on three out of eleven beaches (Figure 5) affected by an algal bloom in 2011, in Algarve [23,33]. This region is the Portugal’s most important “sun and beach” destination. The coast in the western part of Algarve is mostly characterized by flat, near shore rocky bottoms, with small sandy beaches surrounded by sandstone cliffs [22]. Samples were collected on September 26 at Luz and Burgau, and on September 27 at Camilo. Sampling dates coincided with the two last days of bathing prohibition in the affected area, as determined by the local and national authorities [33].

Immediately after collection and during transportation, samples and/or sub-samples were kept cool and in the dark until processing. For each sample, three different sub-samples were made. One was kept fresh for microscopic examination, in 50 mL sterile Falcon tubes. As soon as they arrived at laboratory, microphotographs were taken and cells measured. After homogenizing each sample, 100 mL were transferred into sterile plastic flasks and preserved in Lugol’s solution (Sigma, St. Louis, MO, USA) for microscopic estimation. These sub-samples were kept in the dark until analysis. Finally, for each sample, three different pieces of clumps were aseptically harvested and placed into three sterile 2 mL microcentrifuge tubes. At the laboratory, these sub-samples were centrifuged and the pellets were stored at −20 °C until DNA isolation.

### 4.2. Morphological Analysis and Counting of Microalgae

The morphology of *Ostreopsis* cells present in each sample was examined using a light and fluorescence microscope (Model BX41, Olympus, Hamburg, Germany). Cell dimensions were determined by measuring the dorsoventral (DV) and transversal (width, W) diameters (*n* = 45, 15 in each sample) at 400× magnification, with an image analysis system (Model DP72 microscope digital camera and software Cell B; Olympus, Hamburg, Germany).

For counting purposes, Lugol-fixed subsamples were resuspended and homogenized, and 25 mL was settled in Utermöhl counting chambers. The quantification was done using an inverted microscope (Leitz Labovert, Wetzlar, Germany), according to Lund *et al.* [46]. Microalgae (including cyanobacteria) were identified using standard taxonomic references [47,48,49].

### 4.3. DNA Extraction, PCR Amplification, Cloning and Sequencing

Isolation of genomic DNA from crude material (*i.e.*, floating clumps) collected in each site was performed using the silica bead-based kit ZR Soil Microbe DNA MiniPrep™ (Zymo Research, Orange, CA, USA), following the manufacturer’s instructions. For each sample, DNA was extracted separately from the three replicates (see Subsection 4.1.), and then pooled in equimolar quantities. We tested several primer sets commonly used for the identification of dinoflagellates (e.g., [19,32]), including those developed for the genus- or species- detection and identification of *Ostreopsis* specimens [27]. These primer sets amplify fragments containing different regions of the nuclear rRNA genes and their associated ITS regions (Figure 2). In Table S1 is indicated the polymerase chain reaction (PCR) conditions for each primer set. Main modifications to the PCR protocols were the use of the GoTaq^®^ DNA Polymerase (Promega, Madison, WI, USA) enzyme (~1 × 10^−5^ errors per bp), and the addition of 0.5 μL of 1% (*w*/*v*) BSA (Bovine Serum Albumin; Sigma-Aldrich, St. Louis, MO, USA) in each 20 μL reaction, to circumvent PCR inhibitors.

PCR amplicons were purified from excised agarose gel slices using the Cut&Spin Gel Extraction spin columns (GRISP, Porto, Portugal). Purified amplicons were then cloned into pGEM^®^-T Easy vector (Promega, Madison, WI, USA), and transformed into OneShot^®^ TOP10 chemically competent *E. coli* cells (Invitrogen, Carlsbad, CA, USA) following the manufacturer’s instructions. For each amplification product, white colonies were screened by colony PCR using plasmid-specific primers (pUC/M13), after which several colonies were selected and subsequently grown overnight in LB medium supplemented with 100 mg·mL^−1^ of ampicillin, at 37 °C with orbital shaking. For each amplicon, a small clone library (>30 clones) was established. Plasmid DNA from each selected colony was then isolated using GenElute™ Plasmid Miniprep Kit (Sigma-Aldrich, St. Louis, MO, USA). The inserts (from 100 clones, in total) were sequenced at Macrogen Corp. Europe (Amsterdam, The Netherlands) using pUC/M13 sequencing primers. The genus *Ostreopsis* was confirmed by BLASTn searches in GenBank using the obtained sequences as query, further assessed the existence of chimeras using the Bellerophon program [50], and deposited in GenBank (accession numbers KF612633 to KF612659).

### 4.4. Phylogenetic and Sequence Analysis

Each set of environmental clone sequences obtained in this study was aligned along with the sequences obtained from the BLASTn best hits searches in GenBank as well as with selected *Ostreopsis* spp. sequences retrieved from previous studies [13,19,24,26,31,32,51], covering different geographical regions and biomes. For the ITS1-5.8S-ITS2-28S rDNA multiple sequences alignment, we concatenated the available rRNA gene sequences of the ITS1-5.8S-ITS2 region of a particular isolate with its correspondent sequence of the LSU D1/D2 domain (for a listing, see [19]). Multiple sequence alignments were performed using the ClustalW algorithm implemented in MEGA version 5 software package [52].

jModelTest 2 [53] was used to assess the model of nucleotide substitution that best fitted each aligned dataset under the corrected Akaike’s Information Criterion. The HKY+G model of evolution was selected for the ITS1-5.8S rRNA gene alignment, while the TIM1+G model was selected for the ITS1-5.8S-ITS2-28S rRNA gene alignment. Phylogenetic analyses were performed under the assumptions of the Bayesian inference (BI) using the Bayesian Markov chain Monte Carlo (MCMC) method implemented in the software MrBayes version 3.1.2 [54]. Bayesian searches were performed using a random starting tree, with one cold and seven incrementally heated chains (temperature = 0.2), and were run for 10^7^ generations, with a tree sampling frequency of 100. For each dataset, two independent runs were performed and the consensus phylogeny was built upon confirmation of convergence with the software Tracer 1.4 (burnin = 25%). The same alignments were also used to generate a Maximum-likelihood (ML) tree in MEGA 5 [52], using the previously selected model of nucleotide substitution and assessing node support over 1000 bootstrap replicates. Ambiguously aligned regions were omitted (“complete deletion” option) from the analyses. The topologies retrieved from the ML and BI analyses were evaluated using TreePuzzle 5.2 [55], and the best topology for each dataset was selected according to results from all the test comparisons (1sKH, SH, ELW, and 2sKH). Branch support values derived from the different analyses were compared using TreeGraph 2 [56].

Additionally, analyses of sequence divergence within the environmental clones obtained in this study and among them and their phylogenetically closest relatives were performed in MEGA 5 [52] using the ITS1-5.8S-ITS2-28S rRNA alignment. This dataset was partitioned in genes/spacers for which boundaries were successfully and reliably determined. All positions containing gaps and missing data were eliminated, resulting in a total of 233, 85, and 593 alignment positions for the combined ITS1-5.8S region, the ITS2, and the LSU region, respectively.

### 4.5. ITS2 Secondary Structure Modelling

The ITS2 region of *O.* cf. *ovata* was annotated against homologous sequences of dinoflagellate available from the ITS2-Database version 4 [41].

The secondary structure of the *Ostreopsis* cf. *ovata* ITS2 was predicted using free energy minimization [57], maximum expected accuracy [58], and homology modelling [59] approaches. In free energy minimization, the preferred equilibrium structure is the structure with lowest Gibbs free energy change of folding. The predicted minimum free energy (MFE) structure was calculated using the software RNA structure version 5.5 [60], which implements a nearest-neighbour model to predict the conformational stability of a given structure.

The same software was used to predict the maximum expected accuracy (MEA) structure, a structure that maximizes base-pair probabilities. In this approach, a partition function calculation based on the free energy change nearest-neighbor parameters is used to predict base-pair probabilities as well as probabilities of nucleotides being single-stranded. Finally, we predict the ITS2 secondary structure of *O.* cf. *ovata* by exploiting possible homology to ITS2 available secondary structure in *Alexandrium pseudogoniaulax* from ITS2-Database version 4 [41] and in *Coolia malayensis* from a previous study [37]. We used the ITS2 rRNA of isolate IEO-VGO655 of *A. pseudogoniaulax* (GenBank accession number: AM237416) and of strain CmPL01 of *C. malayensis* (AF244943) as templates. Homology modelling was performed using the algorithm implemented in the ITS2-Database [59] with threshold for helix transfer of 50% and 75%.

## 5. Conclusions

Studies of natural populations of *Ostreopsis* by mean of polyphasic (*i.e.*, involving both phenotypic and genomic traits), culture-independent methods are still scarce. Instead, the majority are based on a culture-dependent approach. Those few existing culture-independent studies have developed [27,61] or used genus- and species-specific primers to detect/identify *Ostreopsis* by PCR [62] or enumerate *Ostreopsis* cells by qPCR [61,63] for monitoring purposes. However, the study of the population’s genetic diversity was not attempted in any case. The culture-independent approach and (phylo)genetic analyses here performed suggest the presence of slightly different genotypes (*i.e.*, strains) of *Ostreopsis* cf. *ovata* within the bloom population, something that deserves to be investigated thoroughly, and in other bloom events. Thus, our findings reinforce the need to apply fingerprinting molecular methods—e.g., classical techniques such as DGGE or SSCP, or preferably NGS techniques-, to ensure that the genetic diversity (*i.e.*, variability at the sub-species level) of *Ostreopsis* is embraced when studying natural populations. We consider that this may lead, in future studies, to a grasp of the genetic diversity and population structuring of *Ostreopsis* spp. in natural communities, and thereof, to a better comprehension of the development and dynamics of blooms of these toxic dinoflagellates.

The successful amplification of a larger fragment of the rRNA gene complex (1052 bp) than those usually used in *Ostreopsis* studies made the accurate determination of the boundaries of the ITS2 possible, a region very often wrongly annotated in sequences deposited in GenBank [36]. We believe that the annotated Algarve’s sequences can be used as a reference for future annotations of *Ostreopsis* ITS2 sequences. Moreover, this >1000 bp fragment shows that more reliable phylogenies and genetic analyses of this genus may be henceforward obtained, even from sequences directly achieved from environmental samples (*i.e.*, eDNA). For instance, this fragment includes several regions with different levels of evolutionary conservation (5.8S, ITS2 and D1/D2 LSU regions), previously shown to be valuable markers to explore patterns of genetic divergence within and among *Ostreopsis* spp. populations [29,31,64]. This study also pointed out an uncommon ITS2 secondary structure in this dinoflagellate genus, which makes this marker unfeasible for estimating dinoflagellates phylogenies with sequence alignments guided by secondary structure predictions [41], whenever *Ostreopsis* sequences are used.

## Supplementary Materials

Supplementary materials can be accessed at: http://www.mdpi.com/2072-6651/7/7/2514/s1.
