# Supplementary material for: Culture-Independent Study of the Late-Stage of a Bloom of the Toxic Dinoflagellate Ostreopsis cf. ovata: Preliminary Findings Suggest Genetic Differences at the Sub-Species Level and Allow ITS2 Structure Characterization
Source: Toxins (Basel). 2015 Jun 30;7(7):2514–33. doi: 10.3390/toxins7072514 (PMC4516926; doi:10.3390/toxins7072514)
Supplement: Supplementary file 1 [file toxins-07-02514-s001.pdf]

Supplementary Information

Table S1. Oligonucleotide primer sets tested for PCR amplification of *Ostreopsis* spp. rRNA genes fragments from total environmental DNA (see also Figure 2).

Primer binding region	Target organisms	Primer name	Antisense primer(s) tested	Sequence (5′→3′)	Reference
28S rRNA gene (D2 region)	Dinoflagellates	D2C [a]	ovataF [a]	CCTTGGTCCGTGTTTCAAGA	[35]
5.8S rRNA gene	*Ostreopsis* spp.	OstreopsisF	OstreopsisR	AAAACGATATGAAGAGTGCAGC	[27]
5.8S rRNA gene	*Ostreopsis* spp.	OstreopsisR	OstreopsisF, ovataF, and siamensisF	CCAGGAGTATGCCTACATTCAA	[27]
ITS1	*Ostreopsis* cf. *ovata*	ovataF [a]	OstreopsisR and D2C [a]	CAATGCTCATGTCAATGATG	[27]
ITS1	*Ostreopsis* cf. *siamensis*	siamensisF	OstreopsisR	TGTTACCATTGCTGAGTTTG	[27]

[a] ovataF/D2C is a new primer combination; the PCR conditions are the same used in [35].

Table S2. Estimates of divergence between *Ostreopsis* cf. *ovata* from different geographical locations, in different sequence regions of the nuclear rRNA gene complex. The number of base substitutions per site from between sequences and the base differences per sequence (within brackets) from between sequences are shown. The analyses involved 10 environmental clone sequences obtained in this study, 18 nucleotide sequences from isolates belonging to the "Mediterranean Sea and West Atlantic (Brazil)" and "East Atlantic (Canary Is.)" subcluster *sensu* Penna *et al.* [19] and 2 sequences from isolates phylogenetically more distant, although included in the *Ostreopsis* cf. *ovata* clade (Figure 3), and here defined as out-group (see also Figure S1). The Mediterranean Sea group comprises 11 sequences from isolates collected in five different locations of the Basin, in six different years. The East Atlantic and the West Atlantic groups include sequences from three and four isolates, respectively. In both cases, the isolates were obtained from a same population. The values highlighted in bold are sequence divergences within the same group.

Group of sequences	Genetic region	Algarve's bloom	Mediterranean	East Atlantic	West Atlantic	Outgroup
**Algarve's bloom**	ITS1-5.8S	**0.003 (0.8)**	-	-	-	-
	ITS2	**0.009 (0.8)**	-	-	-	-
	LSU	**0.009 (5.0)**	-	-	-	-
**Mediterranean**	ITS1-5.8S	0.002 (0.4)	**0.000 (0.0)**	-	-	-
	ITS2	0.006 (0.5)	**0.002 (0.2)**	-	-	-
	LSU	0.005 (2.8)	**0.000 (0.2)**	-	-	-
**East Atlantic**	ITS1-5.8S	0.002 (0.4)	0.000 (0.0)	**0.000 (0.0)**	-	-
	ITS2	0.005 (0.4)	0.001 (0.1)	**0.000 (0.0)**	-	-
	LSU	0.006 (3.7)	0.002 (1.0)	**0.003 (2.0)**	-	-
**West Atlantic**	ITS1-5.8S	0.003 (0.7)	0.001 (0.3)	0.001 (0.3)	**0.002 (0.5)**	-
	ITS2	0.005 (0.4)	0.001 (0.1)	0.000 (0.0)	**0.000 (0.0)**	-
	LSU	0.005 (2.7)	0.000 (0.1)	0.002 (1.0)	**0.000 (0.0)**	-
**Out-group**	ITS1-5.8S	0.168 (27.4)	0.165 (27.0)	0.165 (27.0)	0.167 (27.3)	**0.266 (40.0)**
	ITS2	0.382 (16.6)	0.383 (16.5)	0.381 (16.5)	0.381 (16.5)	**0.678 (24.0)**
	LSU	0.060 (31.1)	0.054 (28.5)	0.055 (28.8)	0.054 (28.5)	**0.077 (39.0)**
**Overall divergences**	**Genetic region**	**within sub-cluster**	**with out-group sequences**			
	ITS1-5.8S	0.002 (0.6)	0.023 (3.9)			
	ITS2	0.009 (0.4)	0.056 (2.6)			
	LSU	0.004 (2.2)	0.011 (5.8)			

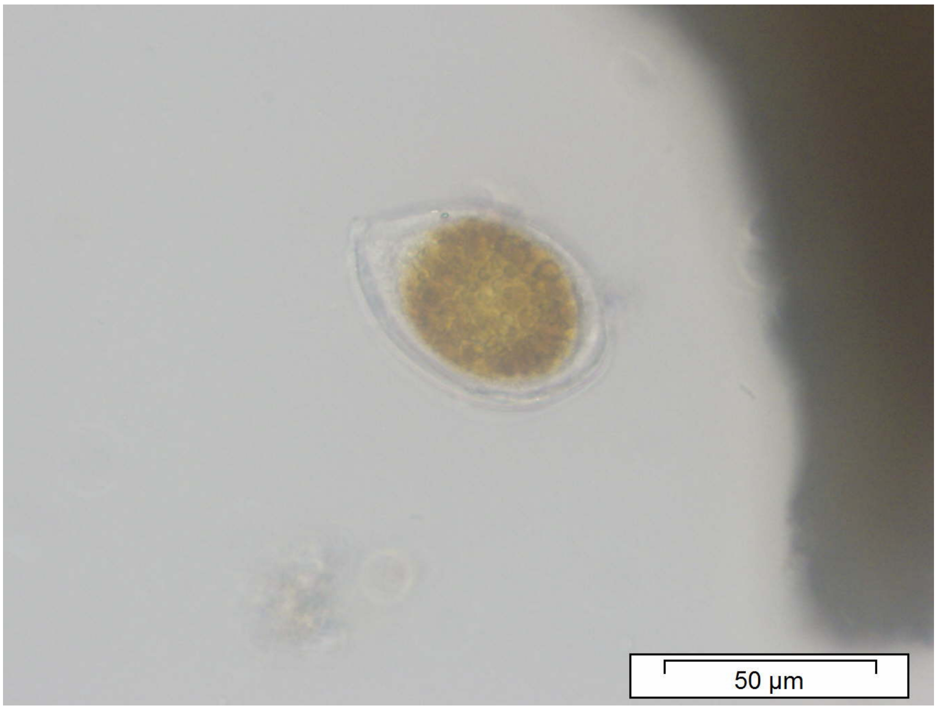

Figure S1. Type A (*sensu* Accoroni *et al*. [25]) thin-walled cyst of *Ostreopsis* cf. *ovata*.

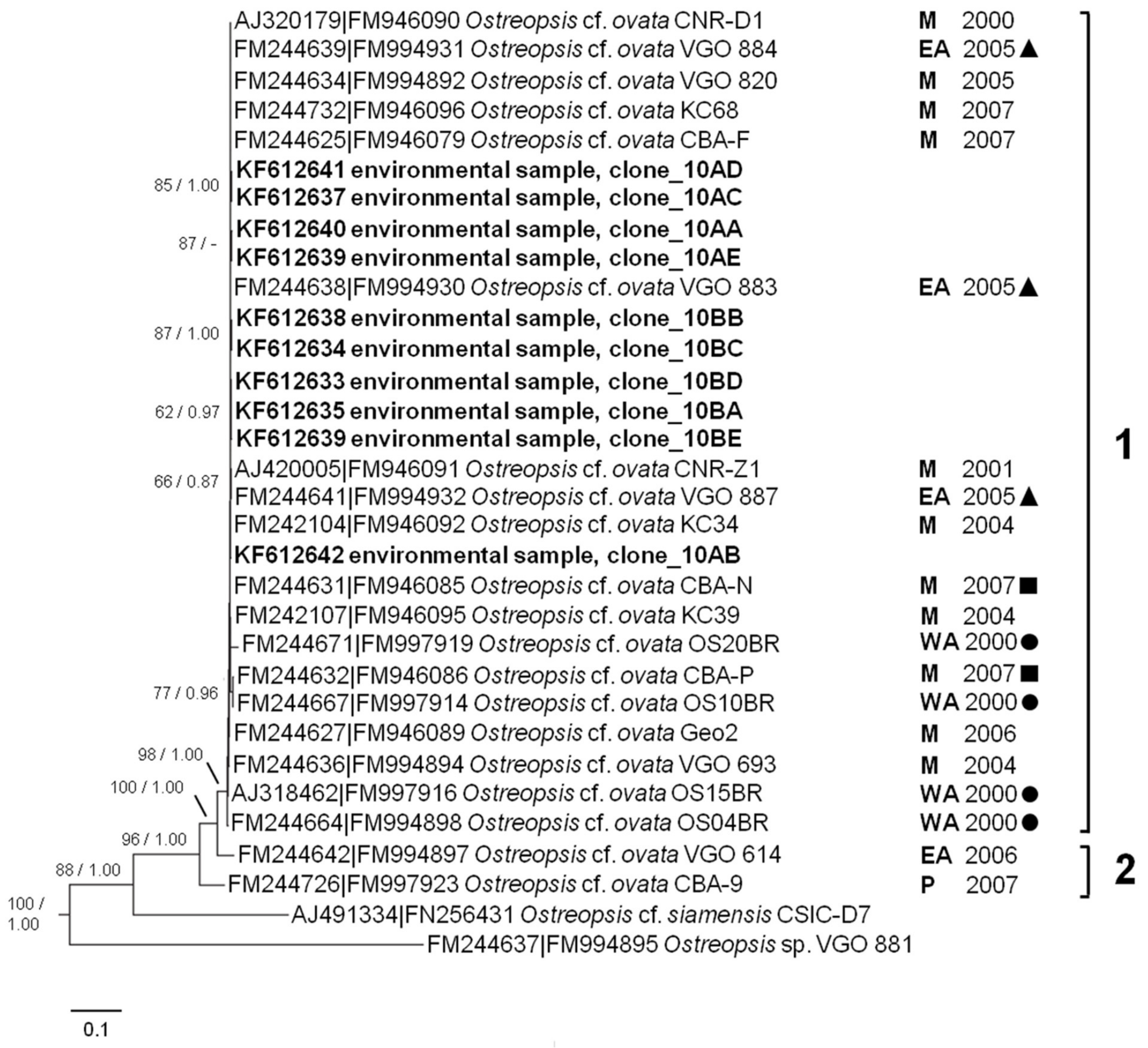

Figure S2. Maximum likelihood tree (−lnL = 7017.75) for ITS1-5.8S-ITS2-LSU rRNA sequences of environmental clones obtained in this study (in bold) and for concatenated sequences of *Ostreopsis* spp. isolates. A total of 1080 unambiguously aligned nucleotide sites were analyzed. Accession numbers before each isolate description refers to sequences of its ITS1-5.8S-ITS2 region and D1/D2 domain of the LSU rRNA gene, respectively. The nodal support values indicated near internal branches were determined by ML and BI methods, respectively. The tree was rooted using *Coolia monotis* VGO 783 (FN256433|AM902747) as outgroup, which was removed for clarity. Scale bar represents 0.1 nucleotide substitutions per site. Legend: **1**, is the sub-cluster of phylogenetically closely-related sequences that were used in the evolutionary divergence analyses; **2**, is the out-group used in the same analyses, as defined in Table S2. **M**, isolates collected in the Mediterranean Sea; **EA**, East Atlantic; **WA**, West Atlantic; **P**, Pacific. Four digit numbers indicate the year of collection. Sequences highlighted with the same symbol (▲, ■, ●) stand for isolates from a same population.
